# SDF1 Polymorphisms Influence Outcome in Patients with Symptomatic Cardiovascular Disease

**DOI:** 10.1371/journal.pone.0161933

**Published:** 2016-09-08

**Authors:** Dominik Rath, Elke Schaeffeler, Stefan Winter, Jens Hewer, Karin Müller, Michal Droppa, Fabian Stimpfle, Meinrad Gawaz, Matthias Schwab, Tobias Geisler

**Affiliations:** 1 Department of Cardiology, University Hospital Tuebingen, Otfried-Mueller-Straße 10, Tuebingen, Germany; 2 Dr. Margarete-Fischer-Bosch Institute of Clinical Pharmacology, Auerbachstrasse 112, Stuttgart, Germany; 3 Department of Clinical Pharmacology, University Hospital Tuebingen, Auf der Morgenstelle 8, Tuebingen, Germany; Universitatsklinikum Hamburg-Eppendorf, GERMANY

## Abstract

**Background:**

SDF1 and its cognate receptors CXCR4 and CXCR7 are involved in myocardial repair and are associated with outcome in cardiovascular patients. Hence, we aimed to investigate clinically significant SDF1 SNPs for their prognostic impact in patients with cardiovascular disease.

**Methods and Results:**

Genotyping for selected SDF1 variants (rs1065297, rs2839693, rs1801157, rs266087, rs266085 and rs266089 was performed in patients (n = 872) who underwent percutaneous coronary intervention. Carriers of variant rs2839693 and rs266089 showed significantly higher cumulative event-free survival compared with non-carriers. All other polymorphisms had no relevant influence on outcome. Multivariate Cox regression analysis showed a significant correlation of these SNPs with cardiovascular outcome after inclusion of clinical and prognostic relevant variables (hazard ratio (HR) 0.51 (95% CI 0.30–0.88), p = 0.015 and [HR 0.51 (95% CI 0.30–0.88), p = 0.016, respectively). In addition, multivariate Cox regression with SDF1 haplotypes revealed a significantly reduced risk for the haplotype carrying the minor alleles of rs2839693 and rs266089 (HR 0.47 (95% CI 0.27–0.84), p = 0.011).

**Conclusion:**

Distinct SDF1 polymorphisms are associated with improved cardiovascular prognosis in CAD patients. Further studies are warranted to validate these results and to better describe the endogenous regeneration potential in carriers of these SNPs. Targeted, genotype guided therapeutic approaches to foster myocardial regeneration and thus cardiovascular prognosis should be evaluated in future.

## Introduction

SDF1 (CXCL-12) is a CXC chemokine and is expressed in a variety of tissues where it acts as a potent chemoattractant for hematopoetic cells.[1,2,3] SDF1 is involved in homing of hematopoietic stem cells to the bone marrow and controlling human- and murine progenitor cell proliferation- and survival.[4,5,6] SDF1 creates a stem cell-attracting environment which possibly results in organ- and tissue repair.[7] Several experimental studies have shown, that high SDF1 levels in ischemic myocardium lead to myocardial protection and improved function after myocardial infarction *in vivo*.[8,9]

Platelets are a relevant source for SDF1. Platelet surface expression of SDF1 is higher in patients with acute coronary syndrome (ACS) compared with those suffering from stable coronary artery disease (CAD).[10,11] Recently, we could demonstrate an influence of SDF-1 platelet surface expression on endothelial progenitor cell recruitment after myocardial ischemia. Furthermore, our group demonstrated an enhanced LVEF% recovery in patients with high platelet SDF-1 expression levels following ACS.[12] SDF1 effects are mediated by its two receptors CXCR4 and CXCR7. Several studies suggest that the SDF1/CXCR4/CXCR7 axis is crucially involved in endothelial progenitor cell recruitment and functional recovery after myocardial ischaemia.[13,14,15] Human platelets constitutively express both CXCR4 and CXCR7. Platelet expressions levels of those two receptors correlate with platelet expression of SDF1. Previously, we showed that platelet surface expression of CXCR7 is elevated in ACS patients when compared to patients with stable CAD. In the same study, high platelet CXCR7 surface expression was associated with improvement of LVEF% after ACS.[15] In a patient collective with symptomatic CAD we found baseline platelet CXCR4 levels to be significantly lower in CAD patients suffering subsequent death or myocardial infarction. Both baseline values of CXCR4 and CXCR7 were significantly associated with all-cause mortality in patients with symptomatic CAD.[16] Several single nucleotide polymorphisms (SNPs) of the SDF1 gene might alter prognosis in cardiovascular patients. There are however little data for SDF1 SNPs in patients with cardiovascular disease. Single nucleotide polymorphisms (SNPs) of the SDF1 gene, including rs1801157, rs2839693, rs1065297, and rs266085 might be associated with the occurrence and prognosis of childhood immune thrombocytopenia (ITP). [17] The SDF1 SNP rs1801157 is associated with premature CAD in Chinese patients.[18] Since SDF1 and its cognate receptors CXCR4 and CXCR7 are involved in myocardial repair and are associated with outcome in cardiovascular patients we aimed to investigate clinically significant SDF1 SNPs for their prognostic impact in patients with cardiovascular disease.

## Methods

### Subjects

SDF1 single-nucleotide polymorphism analysis was performed in 943 consecutive patients with coronary artery disease. All subjects gave written informed consent. Patients were admitted to the department of cardiology of the University of Tuebingen, Germany. The study was approved by the institutional ethics committee (Ethik-Kommission an der Medizinischen Fakultät der Eberhard-Karls-Universität und am Universitätsklinikum Tübingen) (270/2011BO1) and complies with the declaration of Helsinki and the good clinical practice guidelines.[19,20,21]

### Genotyping of SDF1 variants

Genomic DNA was isolated from ethylenediaminetetraacetic acid (EDTA) blood samples using the QIAmp^®^ DNA Blood Mini Kit System (Qiagen, Hilden, Germany). Candidate genetic variants of SDF1 were selected on the basis of previous literature reporting on either clinical importance or functional importance on protein expression profile. The criteria for selection of variants in these genes were a representative allele frequency in Caucasians and clear evidence for functional consequences based on *in vitro/in vivo* data.[22,23,24,25,26] Thus, the following polymorphisms of SDF1 were analysed: rs1065297, rs2839693, rs1801157, rs266087, rs266085 and rs266089. Genotyping for SDF1 variants was performed by matrix-assisted laser desorption/ionization time-of-flight mass spectrometry (MALDI-TOF MS) using the MassARRAY^®^ Compact system (Sequenom, CA, USA) as previously described.[27] Details of primers and assays are available upon request. Approximately 10% of samples within each assay were retyped as a quality control. Study personal assessing outcome was blinded to the case status of the study participants during the entire genotyping process. Minor allele frequencies of SDF1 variants in the study cohort are provided in S1 Table. Linkage disequilibrium (LD) map is shown in S1 Fig.

### Follow-Up

All patients were tracked after initial PCI for clinical events including all cause death, myocardial infarction and ischemic stroke for 360 days after study inclusion. The combined primary endpoint consisted of either time to death, MI or ischemic stroke. Secondary endpoints included the single events of all-cause death, MI and ischemic stroke. 71 patients were lost to follow up (7.5%). The patients lost to follow up did not significantly differ in their baseline characteristics as compared to the group remaining in the study. Follow-up for the primary combined endpoint was performed until first occurrence of one of the pre-defined endpoints. Follow-up was performed by telephone interview and/or review of patients´ charts on readmission by investigators blinded to the results of laboratory testing.

### Statistical analysis

Most statistical analyses were performed using SPSS version 21.0 (SPSS Inc., Chicago, IL, USA). Crosstabulations with Chi-square analysis were performed to analyse baseline characteristics and outcome differences between homozygote carriers of major allele and carriers of minor allele. A two-tailed alpha level <0.05 was considered statistically significant. Unless otherwise stated, p-values were not corrected for multiple testing. Cox regression analysis was applied to compare the association of SDF1 SNPs with the combined endpoint and after adjustment for epidemiological factors influencing cardiovascular outcome. The time-dependent covariate method was used to check the proportional hazard assumption of the model. Survival functions for overall survival and time to death were estimated by Kaplan-Meier curves. The log-rank test was applied to compare survival functions between homozygote carriers of major allele and carriers of minor allele. Package qvalue_2.2.2 of statistical software R- 3.2.3 was used to estimate corresponding q-values, defined as the minimal positive false discovery rate at which the considered log-rank test is called significant. Observed and expected allele and genotype frequencies within populations were compared by means of Hardy—Weinberg equilibrium calculations.[28] Linkage disequilibrium map was created using Haploview (Barrett et al. Bioinformatics 2005). Haplotype analyses were performed with packages haplo.stats_1.7.7 and survival_2.38–3. To be more precise, haplotypes of 6 SDF1-polymorphisms were estimated with function haplo.em. Associations between haplotypes and the combined endpoint were then investigated by weighted uni- and multivariate Cox models, with weights given by the posterior probabilities of haplotype pairs for each patient. Here, rare haplotypes (i.e., with haplotype probability <5%) were combined prior to Cox analysis and haplotype effects were investigated in the dominant model (i.e. combining heterozygote and homozygote carriers of a particular haplotype).

Patients’ characteristics (age, gender, cardiovascular risk factors, co-medication) of the prospective cohort (n = 872) stratified according to SDF1 SNPs are provided in Tables 1 and 2. SDF-1 rs2839693 and rs266089 as well as rs266087 and rs266085 are highly correlated with each other. Thus, we omitted rs266089 and rs266085 from the detailed analysis. Covariates such as cardiovascular risk factors and medication on admission were collected based on patient history and diagnosis during hospital stay. We decided to include common risk factors and medication in a cardiovascular patient collective in our baseline characteristics section. Furthermore, we included important cardiovascular risk factors, clinical factors and medication, that are well known to influence prognosis in our multivariate analysis.

**Table 1:** Baseline characteristics of the complete cohort (n = 872) (hc = homozygote carriers).

Characteristics	All (n = 872)	rs1065297 (n = 870)		p	rs2839693 (n = 870)		p
		(n = 799) hc of major allele	(n = 71) minor allele carriers		(n = 633) hc of major allele	(n = 237) minor allele carriers	
No. of males	662 (70%)	610 (71%)	52 (68%)	0.701	486 (71%)	176 (68%)	0.442
Age (years ± SD)	68 (±12)	68 (±12)	66 (±14)	0.251	68 (±13)	68 (±12)	0.346
**CVRF**							
Arterial hypertension	760 (81%)	702 (81%)	58 (76%)	0.405	551 (81%)	209 (81%)	0.756
Hyperlipidemia	522 (55%)	478 (55%)	43 (57%)	0.635	395 (58%)	126 (49%)	**0.016**
Diabetes mellitus type II	298 (32%)	276 (32%)	22 (29%)	0.837	211 (31%)	87 (34%)	0.275
Smoking	375 (40%)	345 (40%)	29 (38%)	0.835	276 (40%)	98 (38%)	0.568
**Clinical factors**							
Gensini Score	39 (±42)	39 (±43)	36 (±32)	0.520	40 (±43)	36 (±38)	0.209
Atrial fibrillation	195 (21%)	180 (21%)	14 (18%)	0.782	137 (20%)	57 (22%)	0.364
LVEF% normal	447 (47%)	413 (48%)	33 (43%)	0.457	330 (48%)	116 (45%)	0.525
LVEF% mild impairment	241 (26%)	224 (26%)	17 (22%)		166 (24%)	75 (29%)	
LVEF% moderate impairment	155 (17%)	143 (17%)	11 (14%)		113 (17%)	41 (16%)	
LVEF% severe impairment	92 (10%)	81 (9%)	11 (14%)		68 (10%)	24 (9%)	
LVEF% unknown	8 (1%)	4 (0%)	4 (5%)		7 (1%)	1 (0%)	
Renal function (creatinine ±SD)	1.1 (±0.7)	1.0 (±0.6)	1.1 (±1.3)	0.542	1.1 (±0.7)	1.0 (±0.6)	0.902
**Medication on admission**							
Acetyl salicylic acid	495 (52%)	451 (52%)	43 (57%)	0.433	359 (52%)	135 (53%)	0.919
Clopidogrel	108 (11%)	93 (11%)	15 (20%)	**0.018**	80 (12%)	28 (11%)	0.717
Prasugrel	17 (2%)	15 (2%)	2 (3%)	0.571	11 (2%)	6 (2%)	0.461
Ticagrelor	38 (4%)	34 (4%)	4 (5%)	0.568	28 (4%)	10 (4%)	0.880
Oral anticoagulants	80 (8%)	73 (8%)	6 (8%)	0.950	54 (8%)	25 (10%)	0.208
ACE inhibitors	393 (42%)	365 (42%)	28 (37%)	0.386	286 (42%)	107 (42%)	0.915
AT1-receptor antagonists	173 (18%)	158 (18%)	15 (20%)	0.731	117 (17%)	56 (22%)	0.105
Ca-channel inhibitors	180 (19%)	172 (20%)	8 (11%)	**0.049**	131 (19%)	49 (19%)	0.950
Beta blockers	528 (56%)	485 (56%)	41 (54%)	0.764	377 (55%)	149 (58%)	0.475
Statins	425 (45%)	386 (45%)	38 (50%)	0.339	314 (46%)	110 (43%)	0.360
**Reason of admission**							
ACS	350 (40%)	326 (41%)	24 (34%)	0.063	258 (41%)	92 (39%)	0.879
Stable CAD	355 (41%)	314 (39%)	39 (55%)		255 (40%)	98 (41%)	
Other	167 (19%)	159 (20%)	8 (11%)		120 (19%)	47 (20%)	
Missing values for SDF1 polymorphisms		2 (0.2%)			2 (0.2%)		

**Table 2:** Baseline characteristics of the complete cohort (n = 872).

Characteristics	All (n = 872)	rs266087 (n = 872)		p	rs1801157 (n = 872)		p
		(n = 385) hc of major allele	(n = 487) minor allele carriers		(n = 572) hc of major allele	(n = 300) minor allele carriers	
No. of males	662 (70%)	283 (69%)	379 (72%)	0.320	425 (69%)	237 (72%)	0.426
Age (years ± SD)	68 (±12)	69 (±12)	67 (±13)	0.053	68 (±13)	67 (±12)	0.776
**CVRF**							
Arterial hypertension	760 (81%)	340 (82%)	420 (79%)	0.407	499 (81%)	261 (79%)	0.472
Hyperlipidemia	521 (55%)	233 (56%)	289 (55%)	0.793	341 (56%)	181 (55%)	0.953
Diabetes mellitus type II	298 (32%)	124 (30%)	174 (33%)	0.333	184 (30%)	114 (35%)	0.314
Smoking	374 (40%)	163 (39%)	212 (40%)	0.723	237 (39%)	138 (42%)	0.261
**Clinical factors**							
Gensini Score	39 (±42)	40 (±39)	43 (±43)	0.793	37 (±38)	43 (±48)	0.061
Atrial fibrillation	194 (21%)	87 (21%)	108 (20%)	0.450	121 (20%)	74 (22%)	0.561
LVEF% normal	447 (47%)	190 (46%)	257 (48%)	0.840	287 (47%)	160 (48%)	0.929
LVEF% mild impairment	241 (26%)	111 (27%)	130 (25%)		159 (26%)	82 (25%)	
LVEF% moderate impairment	155 (17%)	69 (17%)	86 (16%)		101 (16%)	54 (16%)	
LVEF% severe impairment	92 (10%)	40 (10%)	52 (10%)		62 (10%)	30 (9%)	
LVEF% unknown	8 (1%)	4 (1%)	4 (1%)		4 (1%)	4(1%)	
Renal function (creatinine ±SD)	1.1 (±0.7)	1.1 (±0.7)	1.0 (±0.6)	0.471	1.1 (±0.8)	1.0 (±0.4)	**0.005**
**Medication on admission**							
Acetyl salicylic acid	494 (52%)	206 (50%)	289 (55%)	0.103	318 (52%)	177 (54%)	0.561
Clopidogrel	108 (11%)	47 (11%)	61 (12%)	0.887	67 (11%)	41 (12%)	0.484
Prasugrel	17 (2%)	6 (1%)	11 (2%)	0.458	9 (1%)	8 (2%)	0.290
Ticagrelor	38 (4%)	18 (4%)	20 (4%)	0.684	29 (5%)	9 (3%)	0.137
Oral anticoagulants	79 (8%)	37 (9%)	43 (8%)	0.508	53 (9%)	27 (8%)	0.754
ACE inhibitors	393 (42%)	173 (42%)	222 (42%)	0.908	254 (41%)	141 (43%)	0.677
AT1-receptor antagonists	173 (18%)	64 (15%)	109 (21%)	**0.039**	108 (18%)	65 (20%)	0.420
Ca-channel inhibitors	180 (19%)	78 (19%)	102 (19%)	0.837	120 (20%)	60 (18%)	0.616
Beta blockers	526 (56%)	222 (54%)	306 (58%)	0.167	341 (56%)	187 (57%)	0.726
Statins	424 (45%)	177 (43%)	248 (47%)	0.183	270 (44%)	155 (47%)	0.369
**Reason of admission**							
ACS	350 (40%)	149 (39%)	201 (41%)	0.217	227 (40%)	123 (41%)	0.821
Stable CAD	355 (41%)	170 (44%)	185 (38%)		237 (41%)	118 (39%)	
Other	167 (19%)	66 (17%)	101 (21%)		108 (19%)	59 (20%)	
Missing values for SDF1 Polymorphisms		0 (0%)			0 (0%)		

## Results

Number- and categories of events are shown in Table 3.

**Table 3:** Events and incident rate (IR)/ 100 person years (PY) in the overall cohort (n = 872) (71 patients lost to follow-up).

**Variable**	**Number of events rs1065297** (hc of major allele / minor allele carriers)	**IR/100 PY** (hc major allele / minor allele carriers)	**p**
All cause mortality	N = 51 (48/3)	6.0/4.2	0.540
Myocardial infarction	N = 54 (52/2)	6.5/2.8	0.217
Ischemic stroke	N = 13 (12/1)	1.5/1.4	0.965
Combined endpoint	N = 98 (93/5)	11.6/7.0	0.240
**Variable**	**Number of events rs2839693** (hc of major allele / minor allele carriers)		**p**
All cause mortality	N = 51 (41/10)	6.5/4.2	0.207
Myocardial infarction	N = 54 (45/9)	7.1/3.8	0.072
Ischemic stroke	N = 13 (10/3)	1.6/1.3	0.798
Combined endpoint	N = 98 (80/18)	12.6/7.6	**0.036**
**Variable**	**Number of events rs1801157** (hc of major allele / minor allele carriers)		**p**
All cause mortality	N = 51 (30/21)	5.2/7.0	0.294
Myocardial infarction	N = 54 (35/19)	6.1/6.3	0.901
Ischemic stroke	N = 13 (5/8)	0.9/2.7	0.118
Combined endpoint	N = 98 (61/37)	10.7/12.3	0.459
**Variable**	**Number of events rs266087** (hc of major allele / minor allele carriers)		**p**
All cause mortality	N = 51 (23/28)	6.0/5.7	0.888
Myocardial infarction	N = 54 (19/35)	4.9/7.2	0.171
Ischemic stroke	N = 13 (2/11)	0.5/2.3	0.127
Combined endpoint	N = 98 (38/60)	9.9/12.3	0.255

All patients were tracked after initial PCI. We found a significant difference for the combined endpoint for SDF1 rs2839693 [incidence rate (IR)/100PY 7.6 (minor allele) vs. 12.6 (major allele), p = 0.036] and SDF1 rs266089 [IR/100PY 7.8 (minor allele) vs. 12.5 (major allele), p = 0.049], respectively. Furthermore, we found differences for the secondary endpoint myocardial infarction for the SDF1 SNPs rs2839693 [IR/100PY 3.8 (minor allele) vs. 7.1 (major allele), p = 0.072] and rs266089 [IR/100PY 3.9 (minor allele) vs. 7.1 (major allele), p = 0.086]. These results however failed to be significant. We could not find any significant differences for the SDF1 SNPs rs1065297, rs1801157 and rs266087 (see Table 3). Multivariate Cox regression analysis showed that rs2839693 as well as rs266089 were associated with the primary combined endpoint after adjustment for epidemiological factors [hazard ratio (HR) 0.51 (95% CI 0.30–0.88), p = 0.015] and [HR 0.51 (95% CI 0.30–0.88), p = 0.016] (Table 4).

**Table 4:** Cox Regression analysis for the combined endpoint as dependent variable and clinical factors as covariates in the overall cohort of cardiovascular patients.

Variable	HR rs2839693 (95% CI) **	p	q-value
**CVRF**			
Hypertension	0.82 (0.45/1.50)	0.527	0.184
Hyperlipoproteinaemia	0.78 (0.50/1.22)	0.278	0.166
Smoker	0.77 (0.47/1.28)	0.320	0.176
Diabetes mellitus type II	1.18 (0.76/1.84)	0.459	0.184
**Medication**			
ASA	2.08 (1.24/3.49)	**0.006**	**0.017**
Clopidogrel	1.07 (0.59/1.96)	0.820	0.212
ACE inhibitors	0.69 (0.44/1.10)	0.119	0.1
Beta blockers	1.17 (0.70/1.93)	0.552	0.184
Statins	0.85 (0.53/1.39)	0.524	0.184
**Clinical factors**			
Age	1.06 (1.03/1.08)	**<0.001**	**0.017**
Gender	0.98 (0.61/1.57)	0.975	0.224
LVEF% class*	1.52 (1.24/1.87)	**<0.001**	**0.017**
**Groups**			
SDF1 SNP	0.51 (0.30/0.88)	**0.015**	**0.021**

*numerically coded as 0 (normal), 1 (mild), 2 (moderate), and 3 (severe impairment)

**the SNP rs2839693 is highly correlated with rs266089 (LD = 98, r^2^ = 94), as described in the Supporting Information.

**Fig 1:**
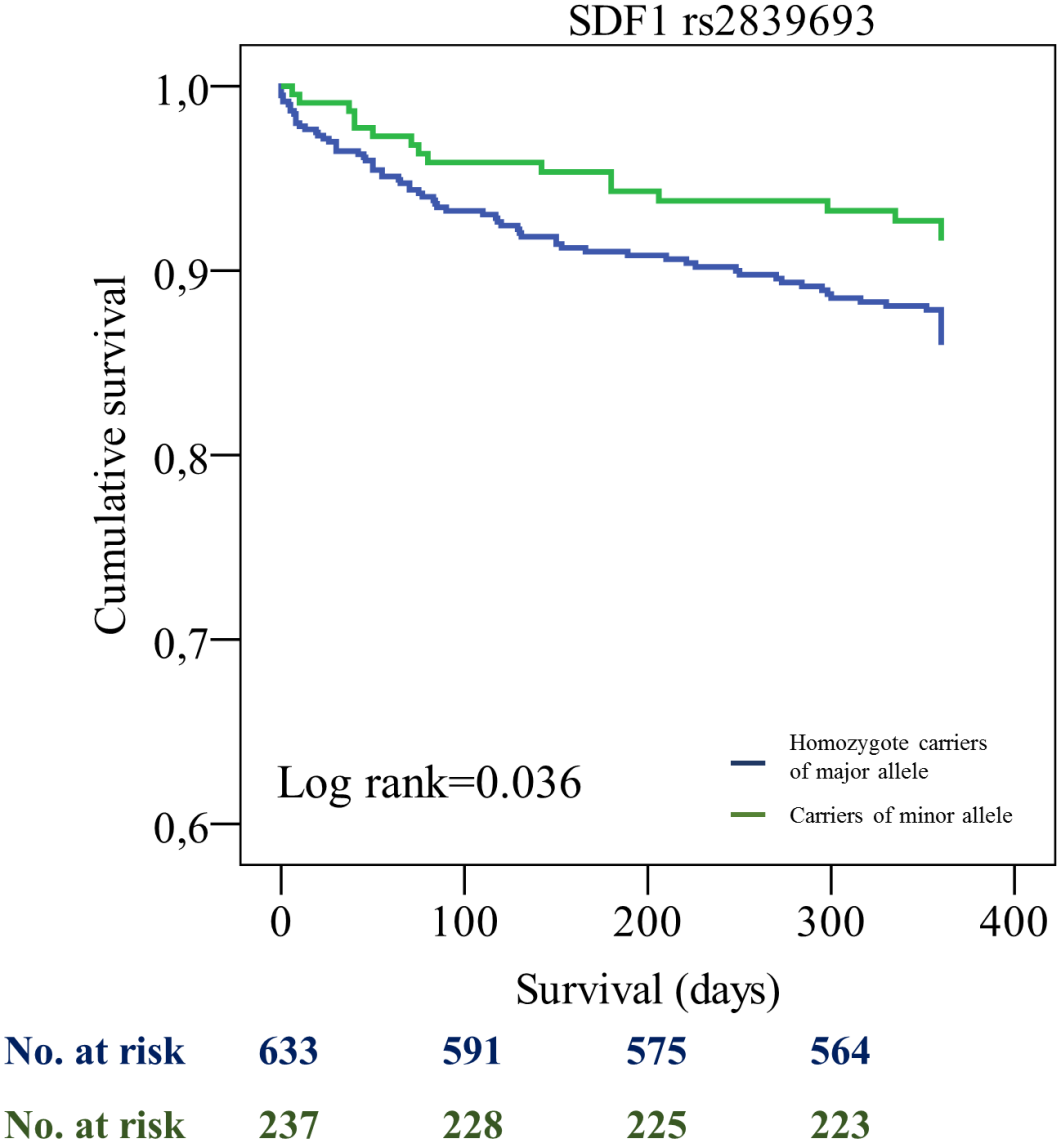
Kaplan-Meier curves showing cumulative survival (combined endpoint all-cause death and/or MI and/or ischemic stroke) stratified according to SDF1 rs2839693 homozygote carriers of major allele and carriers of minor allele. No. at risk: blue = homozygote carriers of major allele, green = carriers of minor allele.

**Fig 2:**
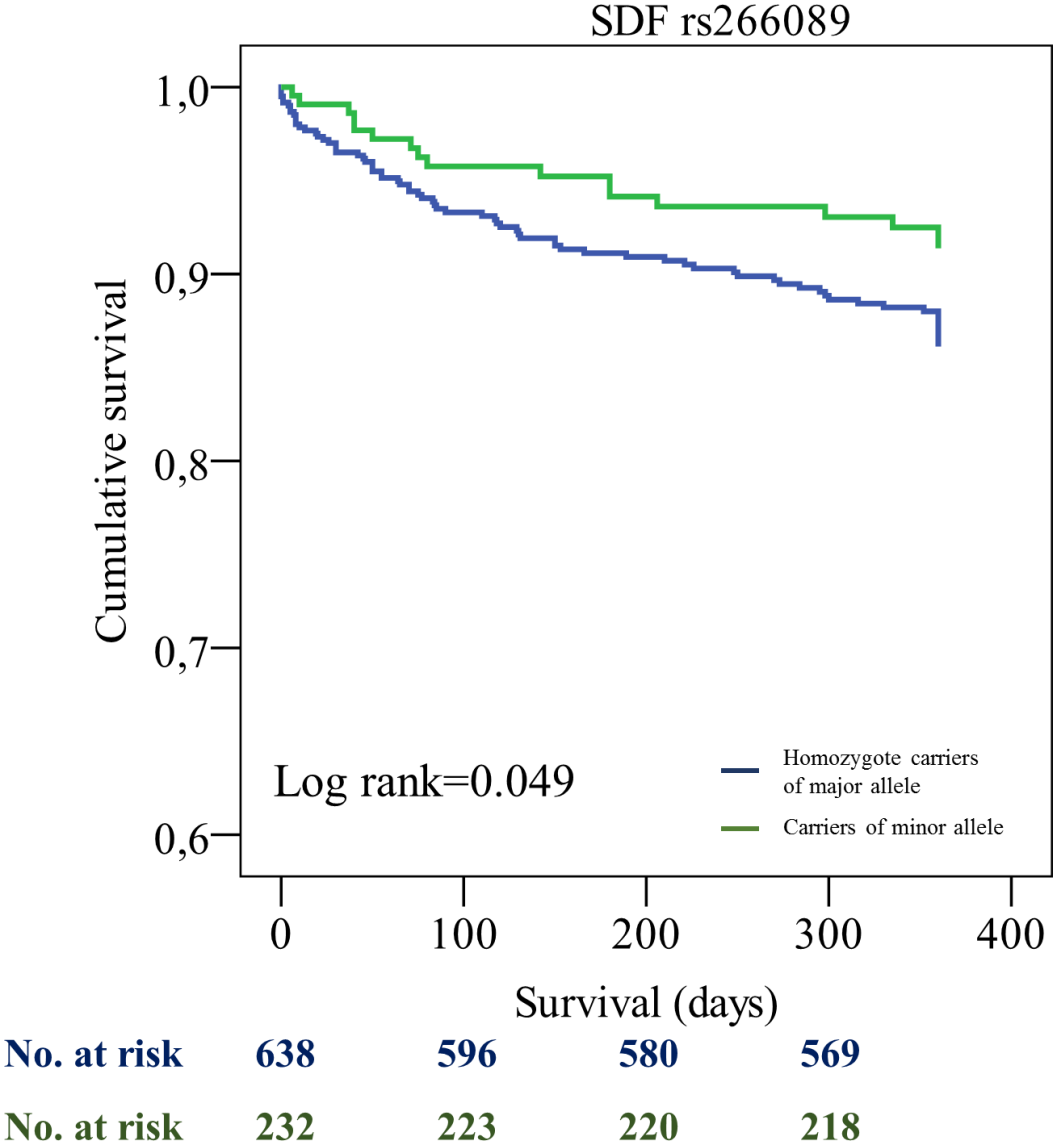
Kaplan-Meier curves showing cumulative survival (combined endpoint all-cause death and/or MI and/or ischemic stroke) stratified according to SDF1 rs266089 homozygote carriers of major allele and carriers of minor allele. No. at risk: blue = homozygote carriers of major allele, green = carriers of minor allele.

**Fig 3:**
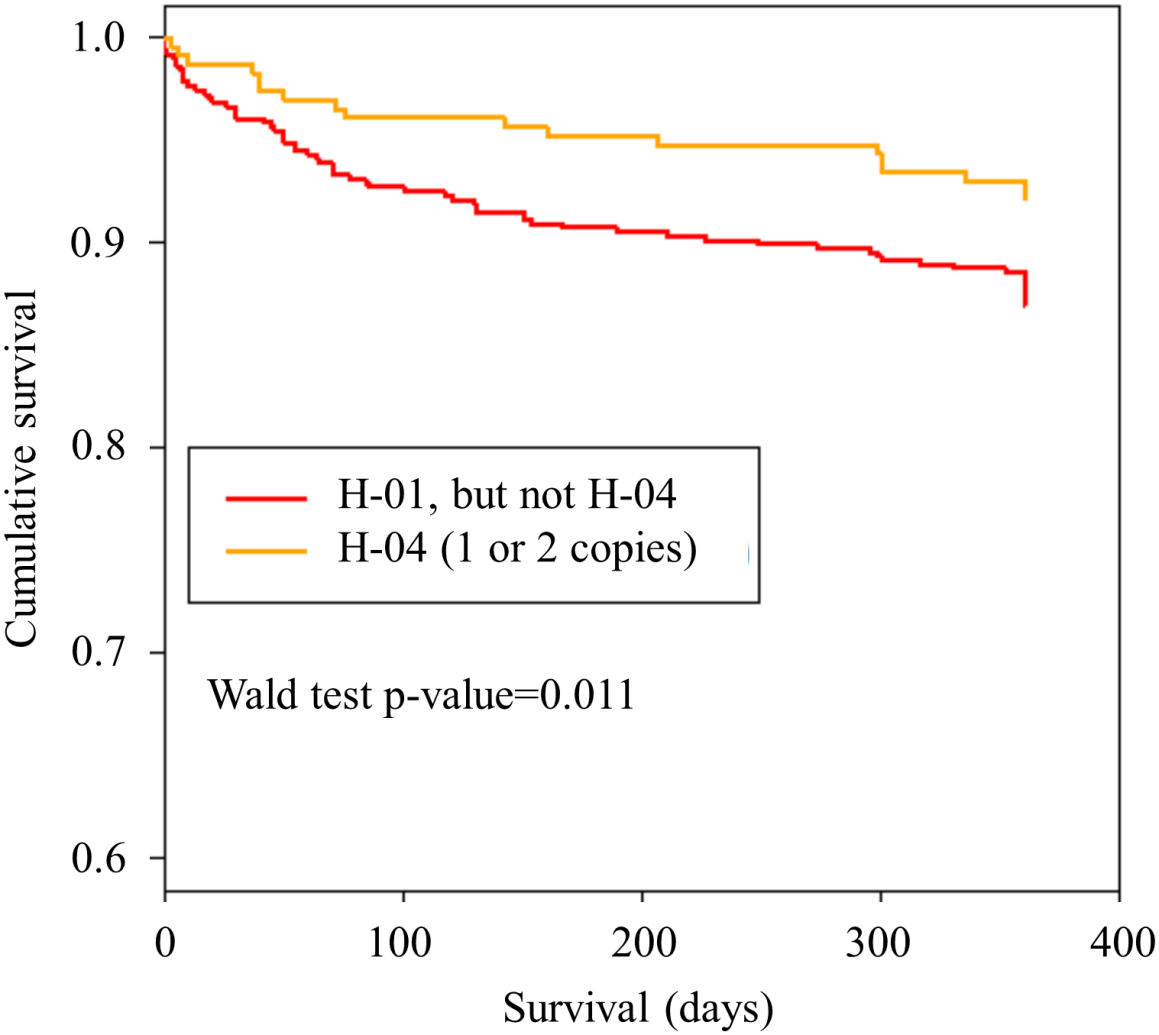
Kaplan-Meier curves showing cumulative survival (combined endpoint all-cause death and/or MI and/or ischemic stroke) stratified according to carriers of reference haplotype H-01 and haplotype H-04 (applying a dominant haplotype model). Wald test p-value refers to our multivariate Cox regression for SDF1 haplotypes (see Table 5). Red = carriers of 1 or 2 copies of H-01, but not H-04, Orange = carriers of 1 or 2 copies of H-04.

Patients who were SDF1 rs2839693 and rs266089 minor allele carriers showed a significantly better event-free survival probability compared to homozygote carriers of major allele (log rank 0.039 and log rank 0.049 for primary combined endpoint) (Figs 1 and 2). Moreover, for the haplotype carrying minor alleles of rs2839693 and rs266089, a borderline association in univariate and a significant effect on the combined endpoint in multivariate Cox regression was revealed (Table 5, Fig 3, and S2 Table).

**Table 5:** SDF1 haplotypes and their effects on the combined endpoint in uni- and multivariate Cox regression.

	SDF1 variants*							Univariate analysis		Multivariate analysis^†^	
Haplotype^‡^	1	2	3	4	5	6	Frequency (%)	HR (95% CI)	p (Wald test)	HR (95% CI)	p (Wald test)
ReferenceH-01							47.3	-	-	-	-
H-02							18.7	0.98 (0.62/1.53)	0.922	0.76 (0.46/1.25)	0.285
H-03							15	1.09 (0.71/1.66)	0.687	1.00 (0.63/1.59)	0.996
H-04							13.8	0.59 (0.35/1.00)	0.052	0.47 (0.27/0.84)	**0.0107**

1 rs1065297, 2 rs1801157, 3 rs266089, 4 rs266087, 5 rs266085, 6 rs2839693

* variants are ordered according to chromosomal position (GRCh38); light grey/dark grey = carriers of major/minor allele

^†^ with covariates as listed in Table 4

^‡^ listed are only haplotypes with frequencies ≥ 5%

## Discussion

The major findings of our study are that the SDF1 SNPs rs2839693 and rs266089 are associated with a better prognosis in patients with cardiovascular disease.

SDF1 is involved in progenitor cell proliferation, traffic, adhesion and regulates cell survival.[2,3,4,5,6,7,29] We have previously shown that platelet SDF1- and CXCR7 expression levels have a significant impact on myocardial function recovery in ACS patients,[12,15] possibly due to a contribution of the SDF1/CXCR4/CXCR7 axis to progenitor cell survival after organ hypoxia.[30] As mentioned before, several experimental studies have suggested that high expression levels of SDF1 in ischemic myocardium result in cardioprotection and improved myocardial function after myocardial infarction *in vivo*.[8,31] Our group developed a bifunctional protein consisting of a SDF1- and a glycoprotein VI (GPVI) domain with high binding affinity to CXCR4, which supports this hypothesis. SDF1-GPVI attracts CXCR4-positive cells, preserves cell survival, enhances endothelial differentiation of bone marrow cells (BMCs) and reveals proangiogenic effects. *In vivo*, administration of the bifunctional protein leads to enhanced recruitment of BMCs, increased capillary density, reduced infarct size, and preserved cardiac function.[32,33] On the other hand, our group could recently show, that high platelet SDF-1 levels might alter outcomes in patients with cardiovascular disease in a negative way (unpublished). SDF1 possesses different functions *in vivo* and SNPs in the SDF1 gene play various roles in many pathophysiologic processes. The gene encoding SDF-1 is located on the human chromosome 10q11.1, which has been previously identified as a susceptibility locus for cardiovascular disease by genome-wide association studies (GWASs).[34,35] Several studies have shown, that certain SDF-1 SNPs might alter SDF-1 protein expression levels.[36,37] However, in our present collective we could neither find a significant alteration of SDF-1 plasma- nor platelet levels within the different SDF-1 haplotypes (data not shown). The lack of alteration in the SDF-1 levels might be explained by the moderate sample size or the inhomogeneity of our study collective. Furthermore, hyperlipidemia is more prevalent in homozygous carriers of major allele than in minor allele carriers, possibly explaining the observations in our current study. Hyperlipidemia is known to be a major cardiovascular risk factor. However, in our multivariate analysis, hyperlipidemia fails to be independently associated with outcome, probably to the moderate sample size and frequent co-medication such as statins. Finally, most traditional cardiovascular risk factors fail to show independent associations with the end point in our study. We believe, that our collective is very heterogeneous and treated in an aggressive way to lower e.g. hypertension, hyperlipidemia and serum glucose levels. In combination with the moderate sample size, this fact might diminish effects of those risk factors on prognosis in our collective. Thus, the mechanisms involved remain speculative and warrant further investigation. Since there are sparse reports dealing with SDF1 SNPs in cardiovascular disease we can hardly compare our results with previous reports. In a study by Feng et al., the SDF1 polymorphism rs1801157 was suggested to be an independent risk factor for development of coronary artery disease.[18] A meta analysis performed by Wu et al suggested that rs1801157 was significantly associated with a decreased risk of myocardial infarction.[38] Furthermore, SDF1 rs1801157 has been demonstrated to influence protein expression levels.[39] There exist controversial data on the influence of the afore mentioned polymorphism on cardiovascular disease, especially when Chinese and Caucasian population are compared.[38] As already mentioned we could not show any differences in plasma SDF-1- or platelet SDF-1 levels for the SNP rs1801157 and we did not investigate incidence of coronary artery disease. Interestingly, we can demonstrate, that minor allele carriers of SNP rs1801157 suffer markedly less from acute coronary syndromes as compared to major allele carriers of the same polymorphism. However, we could not find a significant influence of different SDF-1 polymorphisms on acuity and severity of coronary artery disease i.e. defined by the Gensini Score.[40]

The major clinical implication of the present study is that identification of SDF1 haplotypes (variant rs2839693 and rs266089) might help to characterise cardiovascular patients at high risk for future evens who might benefit from genotype guided targeted therapies. Previously, a number of studies have highlighted the benefits of genotype guided pharmacotherapies in cardiovascular cohorts.[41,42] Although we could not show effects of the investigated SNPs on protein expression and did not evaluate effects on protein function, it is tempting to speculate that therapies targeting SDF-1 mediated effects on cardiovascular regeneration are of potential benefit.

To conclude, this is to the best of our knowledge the first report of the prognostic impact of selected SDF1 SNPs in a large cohort of cardiovascular patients. Thus, we show that SDF1 SNPs rs2839693 and rs266089 are associated with the combined endpoint including death, stroke and myocardial infarction. In addition, multivariate haplotype analysis revealed that the haplotype carrying these two linked SDF1-polymorphisms is significantly associated with the combined endpoint.

There are other additional SDF1 SNPs that might be associated with cardiovascular prognosis. Thus, SDF1 SNPs with rare allele frequency need to be identified by novel sequencing techniques including deep sequencing and independent causal alleles should be evaluated by genome wide approaches.

The present results suggest, that identification of distinct SNPs for SDF1 might help to develop novel pharmacological strategies to foster regenerative processes and to improve cardiovascular outcome. We are aware that these associations warrant confirmation in genome wide association approaches and the effects of guided therapy need confirmation by interventional randomized pharmacogenetic trials.

### Limitations

We are aware that our results are barely hypothesis generating. Our study has certain limitations mainly due to the observational character of the study and the moderate sample size. We did not account for other potential confounders including biomarkers that have previously been associated with outcome in cardiovascular cohorts. Since the study cohort consisted of mostly Caucasian individuals, it might be difficult to generalise the results for other ethnicities. A SNP study should definitely be validated in another cohort. However, a sample size calculation based on a log-rank test yielded that in order to validate the effect of rs266089 with 80% power and for a significance level of 5%, another cohort with at least n = 788 patients would be required. Unfortunately, an additional appropriate cohort of this size for validation was not available. We regard the present study as a first observational study which needs replication in further large scale studies.

## Supporting Information

S1 Fig Linkage disequilibrium map of the SDF1 genomic region including the tested SDF1 variants detected in the total study population. Linkage disequilibrium map was created using Haploview.
(DOCX)

S1 Table Characteristics for SDF1 variants in the complete cohort (n = 943). (DOCX)

S2 Table SDF1 haplotypes and their effects on the combined endpoint after multivariate adjustment for age and gender. (PPTX)

S3 Table Strobe checklist. (DOCX)
