# Supplementary material for: SDF1 Polymorphisms Influence Outcome in Patients with Symptomatic Cardiovascular Disease
Source: PLoS One. 2016 Sep 8;11(9):e0161933. doi: 10.1371/journal.pone.0161933 (PMC5015912; doi:10.1371/journal.pone.0161933)

**Supplementary Fig:**

Linkage disequilibrium map of the *SDF1* genomic region including the tested SDF1 variants detected in the total study population. Linkage disequilibrium map was created using Haploview (Barrett JC, Fry B, Maller J, Daly MJ. Haploview: analysis and visualization of LD and haplotype maps. *Bioinformatics*. 2005)

Coloring corresponds to the standard Haploview interface (D´/LOD shown in the left panel, r^2^ shown in the right panel). Order of SNPs is based on the genomic position (genome reference assembly: GRCh38).


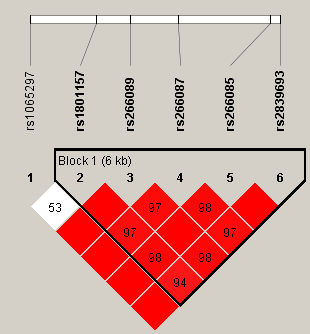

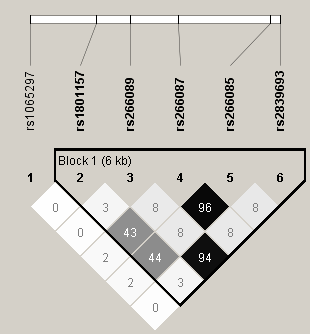

Supplement: S1 Fig — Linkage disequilibrium map was created using Haploview. (DOCX) [file pone.0161933.s001.docx]
