# Supplementary material for: SDF1 Polymorphisms Influence Outcome in Patients with Symptomatic Cardiovascular Disease
Source: PLoS One. 2016 Sep 8;11(9):e0161933. doi: 10.1371/journal.pone.0161933 (PMC5015912; doi:10.1371/journal.pone.0161933)
Supplement: S1 Table — (DOCX) [file pone.0161933.s002.docx]

**Supplementary Table:**

Characteristics for SDF1 variants in the complete cohort (n=943)

| **SDF1 variants** | **Alleles (major/minor)** | **MAF* (%)** | **Missing values (%)** | **HWE p-value^†^** |
| --- | --- | --- | --- | --- |
| rs1065297 | A/G | 4 | 0.2 | 0.396 |
| rs2839693 | C/T | 14.3 | 0.2 | 0.062 |
| rs1801157 | C/T | 19.4 | 0 | 1.000 |
| rs266087 | G/A | 34.1 | 0 | 0.613 |
| rs266085 | C/T | 34.2 | 0.2 | 0.885 |
| rs266089 | G/A | 13.9 | 0.2 | 0.028 |

* Minor allele frequency

† Hardy-Weinberg p-value
