# Supplementary material for: SDF1 Polymorphisms Influence Outcome in Patients with Symptomatic Cardiovascular Disease
Source: PLoS One. 2016 Sep 8;11(9):e0161933. doi: 10.1371/journal.pone.0161933 (PMC5015912; doi:10.1371/journal.pone.0161933)
Supplement: S2 Table — (PPTX) [file pone.0161933.s003.pptx]

## Slide 1
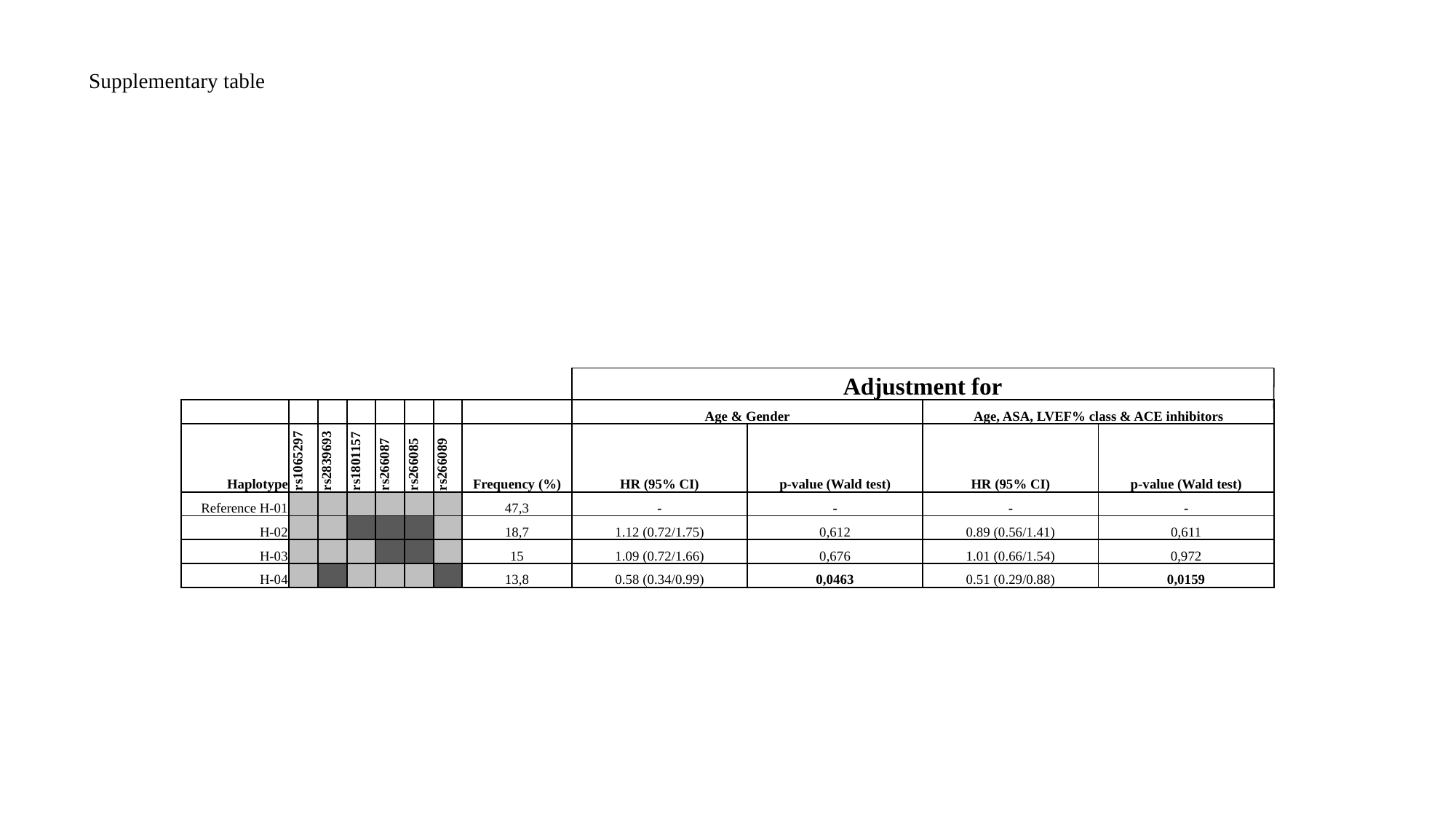

Supplementary table
| | | | | | | | | Adjustment for | | | |
| --- | --- | --- | --- | --- | --- | --- | --- | --- | --- | --- | --- |
| | | | | | | | | Age & Gender | | Age, ASA, LVEF% class & ACE inhibitors | |
| Haplotype | rs1065297 | rs2839693 | rs1801157 | rs266087 | rs266085 | rs266089 | Frequency (%) | HR (95% CI) | p-value (Wald test) | HR (95% CI) | p-value (Wald test) |
| Reference H-01 | 1 | 1 | 1 | 1 | 1 | 1 | 47,3 | - | - | - | - |
| H-02 | 1 | 1 | 2 | 2 | 2 | 1 | 18,7 | 1.12 (0.72/1.75) | 0,612 | 0.89 (0.56/1.41) | 0,611 |
| H-03 | 1 | 1 | 1 | 2 | 2 | 1 | 15 | 1.09 (0.72/1.66) | 0,676 | 1.01 (0.66/1.54) | 0,972 |
| H-04 | 1 | 2 | 1 | 1 | 1 | 2 | 13,8 | 0.58 (0.34/0.99) | 0,0463 | 0.51 (0.29/0.88) | 0,0159 |
